# Retrospective Study of Selective Submandibular Neck Dissection versus Radical Neck Dissection for N0 or N1 Necks in Level I Patients with Oral Squamous Cell Carcinoma

**DOI:** 10.1155/2012/634183

**Published:** 2012-05-28

**Authors:** Yuta Yanai, Tsuyoshi Sugiura, Ikumi Imajyo, Naoya Yoshihama, Naonari Akimoto, Yosuke Kobayashi, Kohei Hayashi, Takahiro Fujinaga, Kanemitsu Shirasuna, Yasuharu Takenoshita, Yoshihide Mori

**Affiliations:** Division of Maxillofacial Diagnostic and Surgical Sciences, Department of Oral and Maxillofacial Surgery, Graduate School of Dental Science, Kyushu University, 3-1-1 Maidashi, Higashi-ku, Fukuoka 812-8582, Japan

## Abstract

*Objective*. To evaluate the efficacy of selective submandibular neck dissection (SMND) in patients with oral squamous cell carcinoma (OSCC) with or without nodal metastasis. *Patients*. From a total of 384 patients with untreated OSCC who underwent radical excision, we identified 229 with clinically N0 necks and 68 with clinically N1 necks in level I. *Main Outcome Measures*. The Kaplan-Meier 5-year regional control and 5-year disease specific survival (DSS) were compared for SMND, radical neck dissection (RND), and modified radical neck dissection (MRND). *Results*. In clinically node-negative necks, the regional control rates were 85.2% with SMND and 83.3% with MRND (*P* = 0.89), and 5-year DSS rates were 86.5% and 87.0%, respectively, (*P* = 0.94). In clinically N1 necks, the regional control rates were 81.3% with SMND and 83.0% with RND (*P* = 0.72), and the DSS rates were 81.3% and 80.0%, respectively, (*P* = 0.94). Type of neck dissection was not significantly associated with regional control or DSS on either univariate or multivariate analysis using Cox's proportional hazard model. *Conclusions*. SMND can be effectively applied in elective and therapeutic management to patients with OSCC that are clinically assessed as N0 or N1 to level I of the neck.

## 1. Introduction

Clinical decisions regarding neck dissection in the treatment of oral squamous cell carcinoma (OSCC) should be based not only on the potential completeness of the resection but also on postoperative functional and cosmetic morbidity. In recent years, selective neck dissection in patients with SCC of the head and neck has been evaluated in terms of patient quality of life, which can be significantly compromised following extensive dissection. However, to our knowledge, little information has been published on determining the appropriateness of selective neck dissection and adequate dissection levels, especially in patients with SCC of the oral cavity. 

Surgical management of neck lymph nodes was classically controlled by radical neck dissection (RND), which resects all of the neck lymph nodes (levels I–V, according to the neck dissection classification of the American Academy of Otolaryngology Head and Neck Surgery [1]). Elective management remains controversial in patients with OSCC who have no clinical evidence of node involvement in the neck. Several studies have asserted the need for elective neck dissection because of the high incidence of occult metastases from OSCC, even in its early stages [2]. Modified radical neck dissection (MRND) is an alternative procedure that avoids dysfunction of the neck region by preserving the accessory nerve, internal jugular vein, and sternocleidomastoid muscle in RND. Supraomohyoid neck dissection (SOHND), a type of selective neck dissection, has gradually become the usual procedure in elective situations [3], with the understanding that OSCC carries a significant risk of metastasizing to cervical lymph nodes in neck levels I, II, and III [4]. Meanwhile, another report recommends a “watchful policy” based on the observation that regional control and overall survival were not significantly different with elective neck dissection versus observation [5]. The conceptual basis for this approach is that lymphatic structures may be considered a defense mechanism against metastasis, so negative nodes should be preserved. Accordingly, we do not perform elective neck dissections that would compromise a patient's quality of life. However, in cases in which the primary tumor invades deep into the tongue or floor of the mouth, level I lymph nodes should be resected as a safety margin even in node-negative necks. For these cases, we have performed submandibular neck dissections (SMNDs). SMND is a superselective neck dissection in only level I necks and is a minimally invasive procedure. We use a transcervical and pull-through approach for resection of the primary tumor and submandibular lymph nodes en bloc.

For clinically node-positive necks, a radical neck dissection (RND) or modified radical neck dissection (MRND) remains the standard surgical treatment [6]. However, selective neck dissection in such cases has been reexamined with increasing understanding of anatomic structures and lymphatic drainage [4, 7]. Several studies have demonstrated the efficacy of SOHND in the clinically N1 neck, with outcomes similar to those of RND and MRND [8, 9]. However, postoperative morbidity, such as shoulder dysfunction, has been reported even with SOHND, leading surgeons to consider less-invasive procedures. At our institution, improvements in imaging have enabled the accurate preoperative diagnosis of metastatic nodes [10]. Therefore, we have performed SMNDs as the initial operation for therapeutic neck dissections in clinically N1 necks with metastasis to level I, followed by careful observation for regional recurrence and immediate salvage treatment if metastasis is detected.

This study aimed to evaluate the efficacy of SMND in elective and therapeutic situations involving OSCC.

## 2. Patients and Methods

We reviewed the medical records of all patients with untreated OSCC who underwent definitive surgery at the Oral and Maxillofacial Surgery, Kyushu University Hospital, between 1989 and 2009 and performed a retrospective cohort study. Patients were followed up at least 5 years after surgery. A total of 384 patients were initially identified. Five patients dropped out within 5 years and were excluded from the study (follow-up rate: 98.7%). Patients with distant metastasis at the initial visit were excluded. The patients who had positive surgical margins at the primary tumor site were excluded from the study because they received extensive salvage surgery or adjuvant chemoradiotherapy. Of these, 229 had clinically defined N0 necks, and 68 had clinically defined N1 necks with metastasis to level I.

All patients underwent radical excision of the primary tumor. Our initial therapeutic approach to cervical metastasis is as follows. (1) Elective neck dissection is not, in principle, performed in clinically defined N0 necks. (2) SMND is performed in N0 necks with a transcervical and pull-through approach for resection of primary tumor and reconstructive surgery. (3) SMND is performed in N1 necks with metastasis only to level I. (4) RND is performed in N1 necks with metastasis to level II or beyond and in N2 necks. Previously, we had performed MRND in N0 necks needing reconstruction (2 above) and RND or MRND in all of the clinically node-positive necks. Most patients who had advanced disease (stage III or IV) received neoadjuvant chemoradiotherapy.

We analyzed the type of neck dissection, use of neoadjuvant or adjuvant therapy, and histopathological factors (e.g., tumor cell differentiation, mode of invasion, and extracapsular spread). The disease was staged according to the International Union Against Cancer (UICC) TNM classification [11]. Classification of neck lymph nodes was done according to the neck dissection classification by the American Academy of Otolaryngology Head and Neck Surgery (AA-OHN) [1]. Tumor cell differentiation was determined using the World Health Organization (WHO) classification scheme [12], and the mode of invasion was determined using the grading system described by Anneroth et al. [13]. Tumor depth in tongue carcinoma was calculated by measuring from the presumed normal mucosal surface to the deepest point of the tumor using magnetic resonance imaging (MRI) and ultrasonography (US). All lymph nodes resected in the surgery were pathologically analyzed. The main outcomes were 5-year regional control and 5-year disease-specific survival (DSS) according to the type of neck dissection, based on the Kaplan-Meier method and log-rank tests. A Cox's proportional hazard model with univariate and multivariate analysis was used to determine variables related to regional control and the DSS. A chi-square test or Fisher's exact test was used to compare the characteristics of each cohort. Statistical significance was defined as a *P* value < 0.05.

The protocol for the research project was approved by a suitably constituted Ethics Committee of Kyushu University. All patients gave informed consent to the individual treatment.

## 3. Results

A total of 297 patients (172 men, 125 women) were included in the analysis. The mean age was 64.3 years (range, 24–87 years). The median followup was 72 months (range, 12–210 months).

### 3.1. Clinically N0 Neck

Among 229 patients with clinically node-negative necks, 110 underwent neck dissection at the initial operation according to the protocol described above, and 119 patients underwent resection of the primary tumor only. The patient characteristics are shown in Table 1. Regional recurrence was present in 16 (14.5%) patients who underwent neck dissection and 21 (17.6%) who did not undergo neck dissection. The 5-year regional control rate was 85.2% in patients who had neck dissections and 82.9% in those who did not (*P* = 0.68), and the 5-year DSS rate was 88.0% and 85.5%, respectively, (*P* = 0.78). 

**Figure 1:**
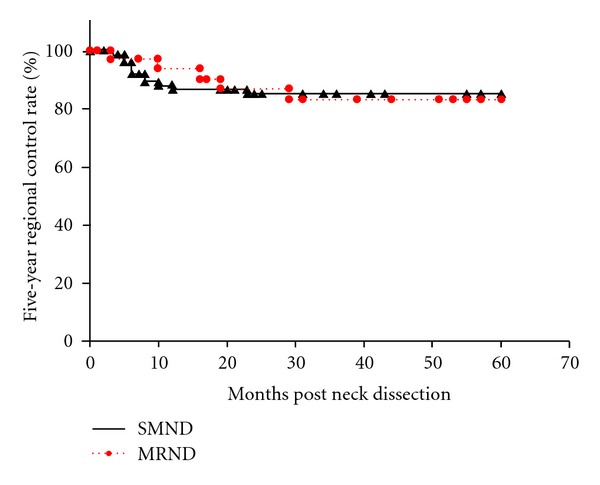
The Kaplan-Meier 5-year regional control rates by type of neck dissection (SMND versus MRND) in clinically N0 necks. SMND: selective submandibular neck dissection; MRND: modified radical neck dissection.

**Figure 2:**
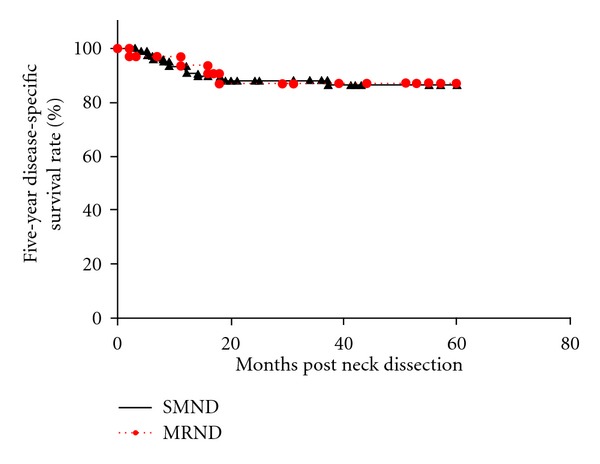
The Kaplan-Meier 5-year disease-specific survival rates by type of neck dissection (SMND versus MRND) in clinically N0 necks. SMND: selective submandibular neck dissection; MRND: modified radical neck dissection.

Of 110 patients who underwent neck dissection, 77 (70%) underwent SMND and 33 (30%) underwent MRND. The patient characteristics are shown in Table 2. Positive nodes were identified histopathologically on the excised specimen in 5 (6.5%) of the 77 patients who underwent SMND and 4 (12.1%) of the 33 who underwent MRND. Among the 4 patients with positive nodes who underwent MRND, 2 had metastases at level I, one at level IIA, and one had multiple metastases at level I and IIA. No patient had evidence of “skip metastasis” to level III or IV or metastasis to level IIB or V. Occult metastases were present in 9 (8.2%) of all 110 patients, and the remaining 101 (91.8%) patients had histopathologically node-negative disease. Regional recurrences were documented in 11 (14.3%) patients who underwent SMND and 5 (15.1%) who underwent MRND, resulting in 5-year regional control rates of 85.2% and 83.3%, respectively, (*P* = 0.89) (Figure 1). Most of the nodal metastases in patients who underwent SMND were in the ipsilateral neck (8 at level IIA and 1 with multiple metastases at level IIA and III), while 2 were present at level I of the contralateral neck. Most of the patients with nodal metastases experienced regional recurrence within several months to a year. Only 1 patient who underwent MRND had regional recurrence in the ipsilateral neck (i.e., intraglandular parotid node, outside of the neck dissection), while 4 had recurrence in the contralateral neck: 2 at level I, 1 at level IIA, and 1 at level IIA and III. The 5-year DSS rate was 86.5% with SMND and 87.0% with MRND (*P* = 0.94) (Figure 2). On multivariate analysis (Table 3), regional control was negatively associated with pathological nodal stage (node-positive) and extracapsular spread and positively associated with administration of neoadjuvant chemoradiotherapy. DSS was negatively associated with clinical tumor stage, pathological nodal stage, extracapsular spread, and neoadjuvant chemoradiotherapy. Regional control and survival were not significantly associated with type of neck dissection on either univariate or multivariate analysis. 

### 3.2. Clinically N1 Neck

**Figure 3:**
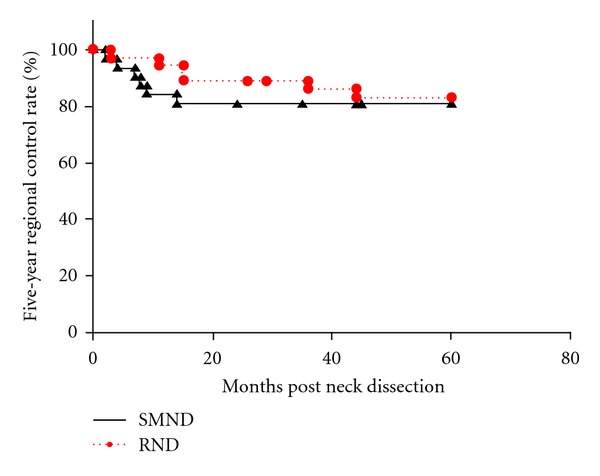
The Kaplan-Meier 5-year regional control rates by type of neck dissection (SMND versus RND) in clinically N1 necks. SMND: selective submandibular neck dissection; RND: radical neck dissection.

**Figure 4:**
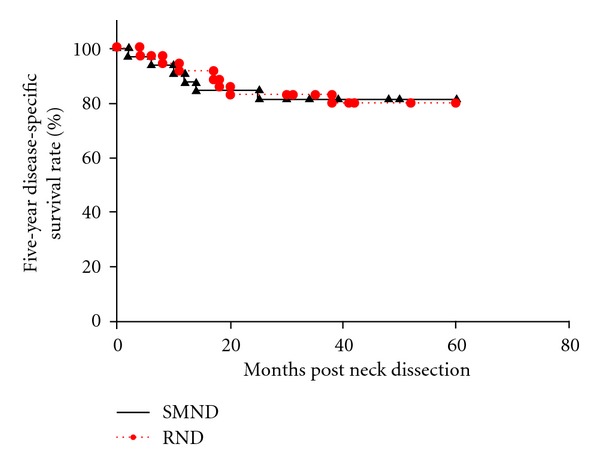
The Kaplan-Meier 5-year disease-specific survival rates by type of neck dissection (SMND versus RND) in clinically N1 necks. SMND: selective submandibular neck dissection; RND: radical neck dissection.

Among 68 patients who had clinically N1 necks with metastasis to level I, 32 underwent SMND and 36 underwent RND. The patient characteristics are shown in Table 2. Of the 32 patients who underwent SMND, 5 (including the cases of complete response on preoperative chemoradiotherapy) had histopathologically node-negative necks, and the other 27 had histopathologically positive necks, including 3 patients with multiple metastases at level I. Of the 36 patients who underwent RND, 6 had histopathologically N0 necks, 25 had pN1 (at level I) necks, and 5 had pN2b neck. Among these 5 patients, 2 had multiple nodal metastases at level I, 2 at level I and sublevel IIA, and one at level I, IIA, III. Eight (11.8%) of all 68 patients had occult nodal metastases, and no patients had skip metastasis to level III or IV or metastases to level IIB or IV. Six (18.8%) patients who underwent SMND and 6 (16.7%) who underwent RND had regional recurrence, leading to 5-year regional control rates of 81.3% and 83.0%, respectively, (*P* = 0.72) (Figure 3). Of the 6 patients who underwent SMND followed by regional recurrences, 4 had recurrences in the ipsilateral neck: 3 at level IIA and 1 at level IIA and III. The other 2 had recurrences at level I in the contralateral neck. Most of them experienced regional recurrences within 1 year. Of the 6 patients who underwent RND followed by regional recurrences, 1 had a recurrence in the parapharyngeal space of the ipsilateral neck, while 5 had recurrences in the contralateral neck: 2 at level I, 2 at level IIA, and 1 at level IIA, III. The 5-year DSS rate was 81.3% after SMND and 80.0% after RND, respectively, (*P* = 0.94) (Figure 4). On multivariate analysis, as in N0 neck, regional control and DDS were negatively associated with pathological nodal stage (pN2) and extracapsular spread and positively associated with administration of neoadjuvant chemoradiotherapy. The type of neck dissection did not significantly correlate with regional recurrence and DDS on either univariate or multivariate analysis (Table 4). Postoperative limited shoulder mobility was present in 11 (30.6%) of the 36 patients who underwent RND.

## 4. Discussion

Indications for elective neck dissection and its appropriate extent in patients with OSCC remain controversial. Elective neck dissection has become more widely used due to the possibility of occult metastases to cervical lymph nodes [2]. Weiss et al. reported that elective treatment of the neck is warranted if the probability of occult metastases is determined to be greater than 20% using decision analysis [14]. The reported probability of occult metastasis has ranged widely from 8 to 48.2%, based on a variety of methods such as routine hematoxylin-eosin staining and immunohistochemical or molecular analysis [2, 15, 16]. Although several predictors of occult metastases have been assessed (e.g., tumor depth, tumor cell differentiation, mode of invasion, and expression of molecular markers) [17–19], micrometastases remain difficult to detect preoperatively. Thus, elective neck dissection has been performed without an established method for assessing the probability of occult metastases. Furthermore, several studies have demonstrated that regional control and overall survival are similar in elective neck dissection and observation, as well as in salvage treatment for regional recurrence [20–22].

Based on these various research findings and practices, one must carefully consider and discuss with the patient whether or not to perform elective neck dissection in clinically N0 necks, because such an approach could constitute overtreatment and unnecessarily compromise the patient's quality of life. To assist in these decisions, we have used power Doppler US and enhanced computed tomography (CT) to examine patients for metastatic lymph nodes. These tests have proven diagnostically sensitive and specific [10]. Additionally, the incidence of occult metastases was 12.1% in our series (Table 2), even in patients treated with elective radical neck dissection. This rate is below the suggested probability of occult metastases (20%) described by Weiss et al. [14]. For this reason, we do not perform elective neck dissections at present. We have performed salvage neck dissection when metastatic nodes are detected. Therefore, careful followup, including frequent imaging tests with US as the primary imaging modality, is needed to detect regional recurrence immediately. We recommend US because it involves less radiation exposure than CT imaging. As mentioned inSection 2,we have performed elective neck dissection only in cases of a transcervical approach with reconstructive surgery. Accordingly, although we could not assess the significance of elective neck dissection in all patients, regardless of the need for reconstructive surgery, we found that regional control and DSS were equivalent in patients who underwent neck dissection and those who underwent tumor resection without neck dissection, followed by observation. Consequently, elective neck dissection does not contribute to improved outcome, and our “watchful” treatment strategy for the clinically N0 neck seems justified. 

Several studies, including multi-institutional prospective randomized studies, have demonstrated that regional control and survival in the clinically negative neck were similar in patients treated with SOHND and MRND. Thus, SOHND has gradually gained acceptance in elective situations [3]. However, some authors have recommended extended SOHND, including level IV, due to the high incidence of skip metastasis to levels III and IV [2, 23] and lymphatic drainage to level II–IV from tongue carcinoma [7]. In addition, some authors have argued that sublevel IIB may be preserved in elective neck dissections, because OSCC rarely metastasizes to this sublevel [24]. As with extended SOHND, ordinary SOHND including sublevel IIB may cause dysfunction of the accessory nerve, as well as phrenic neuropathy, chyle fistula, and cosmetic disturbance [2]. In this way, even “selective” elective neck dissection may be overly invasive for patients with clinically negative necks. We had performed MRND as elective neck dissection along with resection of primary tumors in the past. At present, however, we perform SMND, which is more selective and less invasive than SOHND, based on the conclusion that elective neck dissection is unnecessary. We found that regional control and DDS were equivalent after SMND and MRND regardless of the primary site or histopathological malignancy grade; in addition, no patients in our series had evidence of skip metastasis to level III or IV. Most regional recurrence in patients with SMND was detected at level IIA in the ipsilateral neck within 1 year. Although these patients may have had occult metastases outside the SMND, 7 of 11 patients with regional recurrence underwent successful salvage surgery. In addition, 101 of 110 patients with clinically N0 necks were confirmed on histopathological examination to be node negative; therefore, the diagnostic accuracy of imaging tests for detecting metastatic nodes was considered excellent. Thus, we do believe that SMND is acceptable as an elective neck dissection to accompany primary tumor resection.

Therapeutic selective neck dissection in the clinically positive neck remains more controversial. Several studies have reported that regional control with therapeutic SOHND in clinically N1 or N2 necks was not very different from that with MRND [8, 9]. In a multi-institutional prospective study, the authors recommended SOHND for all patients with clinically N1 necks [25]. On the other hand, another study demonstrated that RND should be performed in clinically positive necks because of the risk of skip metastasis to levels III and IV and of incomplete dissection with SOHND [6, 23]. However, more extensive neck dissection causes more serious morbidity, as Krause reported that 31% of patients with RND had severe shoulder dysfunction (72% of patients had some grade of dysfunction) [26]. We assessed the efficacy of SMND in clinically N0 necks, as noted earlier, and had good results following salvage treatment for patients with regional recurrence. Based on these findings, in recent years we have performed SMND even as a therapeutic neck dissection in clinically N1 necks with metastasis only to level I. In the current series, regional control and DDS in this situation were similar for both SMND and RND, and the outcomes were considered acceptable. The primary tumor site and histopathological malignancy grade did not significantly correlate with outcome on multivariate analysis. This further indicated that SMND was appropriate for all patients with clinically N1 necks with metastasis to level I. Most regional recurrence in patients who underwent SMND, as well as in patients with N0 necks, was detected at sublevel IIA in the ipsilateral neck early in followup. Among 6 patients who had regional recurrence, 4 underwent successful salvage treatment. The fact that all the regional recurrences were detected in dissection levels of SOHND suggests that SMND is inferior to SOHND with regard to completeness of operation. Nevertheless, we were able to avoid overly invasive surgery and prevent postoperative shoulder syndrome, the main residual disability that significantly reduces quality of life in patients without regional recurrences. Hence, SMND should be planned only if the patients can be followed up by frequent and strict postoperative management. We believe that therapeutic SMND can be applied to carefully chosen patients with OSCC, given the low incidence of postoperative functional or cosmetic disorders and excellent outcomes. In addition, 60 of 68 patients with clinically N1 necks were histopathologically confirmed to have N0 (including complete responses to preoperative therapy) or N1 necks. Our strategy for the application of selective neck dissection may also be supported by accurate preoperative diagnosis and immediate salvage treatment on regional recurrence.

## 5. Conclusion

In summary, this is the first paper evaluating the validity of new superselective neck dissection. SMND is an effective treatment for OSCC with N0 necks or N1 necks with metastasis to level I, when careful followup is provided to detect regional recurrence immediately. Cervical metastasis is the most crucial prognostic factor in the treatment of OSCC. 

Therefore, further prospective randomized studies should be planned based on this retrospective study.

## Figures and Tables

**Table 1:** Characteristics of patients (elective neck dissection versus observation).

	Elective neck dissection	Observation	*P* value
Primary tumor site			0.565
Tongue	41 (37.3%)	43 (36.1%)	
Lower gum	44 (40.0%)	38 (31.9%)	
Upper gum	10 (9.1%)	13 (10.9%)	
Buccal mucosa	9 (8.2%)	14 (11.8%)	
Oral floor	3 (2.7%)	8 (6.7%)	
Others	3 (2.7%)	3 (2.5%)	
Clinical primary tumor stage			
cT1/2	78 (70.9%)	86 (72.3%)	0.467
cT3/4	32 (29.1%)	33 (27.7%)	
Tumor differentiation			
Poorly differentiated	7 (6.4%)	6 (5.0%)	0.788
Moderately differentiated	32 (29.1%)	39 (32.8%)	
Well differentiated	71 (64.5%)	74 (62.2%)	
Neoadjuvant chemoradiotherapy	38 (34.5%)	42 (35.3%)	0.508

Total	110	119	

**Table 2:** Characteristics of patients who underwent neck dissection (clinically N0 neck/clinically N1 neck).

Type of neck dissection	Clinically N0 neck (110)			Clinically N1 neck (68)		
	SMND (77)	MRND (33)	*P * value	SMND (32)	RND (36)	*P* value
Primary tumor site						0.825
Tongue	28 (36.4%)	13 (39.4%)	0.923	14 (43.8%)	13 (36.1%)	
Lower gum	31 (40.3%)	13 (39.4%)		11 (34.4%)	14 (38.9%)	
Upper gum	7 (9.1%)	3 (9.1%)		3 (9.4%)	4 (11.1%)	
Buccal mucosa	6 (7.8%)	3 (9.1%)		1 (3.1%)	3 (8.3%)	
Oral floor	2 (2.6%)	1 (3.0%)		3 (9.4%)	2 (5.6%)	
Others	3 (3.9%)	0 (0.0%)		0 (0.0%)	0 (0.0%)	
Clinical tumor stage						
cT1/2	55 (71.4%)	23 (69.7%)	0.513	10 (31.3%)	9 (25.0%)	0.381
cT3/4	22 (28.6%)	10 (30.3%)		22 (68.7%)	27 (75.0%)	
Tumor differentiation						
Poorly differentiated	5 (6.5%)	2 (6.1%)	0.982	2 (6.3%)	2 (5.6%)	0.991
Moderately differentiated	22 (28.6%)	10 (30.3%)		9 (28.1%)	10 (27.8%)	
Well differentiated	50 (64.9%)	21 (63.6%)		21 (65.6%)	24 (66.7%)	
Mode of invasion						
Grade 1–3	58 (75.3%)	23 (69.7%)	0.348	22 (68.8%)	23 (63.9%)	0.435
Grade 4	19 (24.7%)	10 (30.3%)		10 (31.2%)	13 (36.1%)	
Neoadjuvant chemoradiotherapy	26 (33.8%)	12 (36.4%)	0.479	19 (59.4%)	19 (52.8%)	0.325
Pathological nodal stage			0.603			
pN0	72 (93.5%)	29 (87.9%)		5 (15.6%)	6 (16.7%)	0.828
pN1	4 (5.2%)	3 (9.1%)		24 (75.0%)	25 (69.4%)	
pN2b	1 (1.3%)	1 (3.0%)		3 (9.4%)	5 (13.9%)	
Extracapsular spread	1 (1.3%)	2 (6.1%)	0.214	3 (9.4%)	4 (11.1%)	0.567
Adjuvant chemoradiotherapy	5 (6.5%)	4 (12.1%)	0.264	2 (6.3%)	4 (11.1%)	0.395

SMND: selective submandibular neck dissection; RND: radical neck dissection; MRND: modified radical neck dissection.

**Table 3:** Cox's proportional hazard model with univariate and multivariate analysis in clinically N0 necks.

	Regional control						Disease-specific survival				
	Univariate			Multivariate			Univariate			Multivariate	
	Hazard ratio (95% CI)	*P* value		Hazard ratio (95% CI)	*P* value		Hazard ratio (95% CI)	*P* value		Hazard ratio (95% CI)	*P* value
Type of neck dissection MRND/SMND	1.061 (0.381–2.935)	0.913					0.986 (0.337–2.886)	0.980			
Clinical tumor stage T3, 4/T1, 2	1.896 (0.742–2.885)	0.204					3.048 (1.105–8.043)	**0.031**		14.949 (8.643–21.352)	**0.002**
Primary tumor site Tongue	1.113 (0.412–2.080)	0.857					1.219 (0.539–3.052)	0.794			
Tumor depth (Tongue) ≧4 mm	1.687 (0.659–4.260)	0.465					2.386 (0.935–4.777)	0.061			
Poorly or Moderately differentiated	1.061 (0.403–2.446)	0.913					1.490 (0.530–4.187)	0.449			
Mode of invasion Grade 4	2.074 (0.815–4.243)	0.148					1.975 (0.871–4.130)	0.350			
Neoadjuvant chemoradiotherapy	0.277 (0.107–0.825)	**0.045**		0.136 (0.050–0.469)	**0.049**		0.269 (0.076–0.953)	**0.042 **		0.412 (0.150–0.924)	**0.032 **
Pathological nodal stage pN(+)	5.101 (2.470–10.172)	**0.003**		3.542 (1.447–7.122)	**0.027**		4.081 (1.299–12.816)	**0.016 **		6.541 (2.463–9.257)	**0.014 **
Extracapsular spread	5.095 (1.383–18.461)	**0.031**		31.333 (13.443–75.119)	**0.005**		5.487 (2.238–14.315)	**0.025 **		15.915 (11.440–27.967)	**0.024 **
Adjuvant chemoradiotherapy	2.589 (0.643–8.849)	0.137					2.806 (0.792–9.942)	0.110			

SMND: selective submandibular neck dissection; MRND: modified radical neck dissection; CI: confidence interval.

**Table 4:** Cox's proportional hazard model with univariate and multivariate analysis in clinically N1 necks.

	Regional control						Disease specific survival				
	Univariate			Multivariate			Univariate			Multivariate	
	Hazard ratio (95% CI)	*P* value		Hazard ratio (95% CI)	*P* value		Hazard ratio (95% CI)	*P *value		Hazard ratio (95% CI)	*P* value
Type of neck dissection RND/SMND	0.889 (0.289–2.412)	0.838		0.897 (0.224–2.263)	0.962		0.989 (0.346–3.086)	0.948		0.898 (0.209–2.225)	0.796
Clinical tumor stage T3, 4/T1, 2	2.857 (0.794–5.941)	0.086					3.112 (0.990–10.434)	**0.040**		2.465 (1.107–5.261)	0.071
Primary tumor site Tongue	1.129 (0.443–2.676)	0.877					1.024 (0.497–2.311)	0.798			
Tumor depth (tongue) ≧4 mm	1.857 (0.665–3.838)	0.101					1.414 (0.736–4.109)	0.385			
Poorly or moderately differentiated	2.238 (0.733–4.496)	0.163					3.212 (0.876–7.769)	0.073			
Mode of invasion Grade 4	3.250 (1.805–7.727)	**0.041 **		2.966 (0.849–7.330)	0.760		3.814 (1.018–9.012)	**0.036 **		3.145 (1.883–7.516)	0.240
Neoadjuvant chemoradiotherapy	0.263 (0.092–0.706)	**0.045 **		0.195 (0.087–0.451)	**0.013 **		0.312 (0.152–0.942)	**0.039 **		0.232 (0.090–0.596)	**0.018 **
Pathological nodal stage pN2	3.750 (1.825–7.586)	**0.031 **		6.869 (2.681–14.011)	**0.022 **		5.847 (1.888–10.719)	**0.005 **		6.989 (2.428–12.111)	**0.004 **
Extracapsular spread	6.224 (2.484–12..234)	**0.002 **		11.342 (5.880–20.419)	**<0.001**		9.868 (4.510–18.225)	**<0.001**		12.767 (6.926–19.446)	**<0.001**
Adjuvant chemoradiotherapy	1.743 (0.454–4.379)	0.473					2.637 (0.720–9.499)	0.129			

SMND: selective submandibular neck dissection; RND: radical neck dissection; CI: confidence interval.
